# Preventing a Scarring Start into the Labor Market: Integration Strategies for Young Persons with Disabilities

**DOI:** 10.1007/s10926-023-10111-9

**Published:** 2023-03-20

**Authors:** Nancy Reims, Angela Rauch, Ulrich Thomsen

**Affiliations:** Institute for Employment Research (IAB), Regensburger Str. 100, 90478 Nuremberg, Germany

**Keywords:** Vocational rehabilitation, Active labor market programs, Germany, Regression, Program allocation, Labor market structure, School-to-work-transition

## Abstract

**Purpose:**

Sociodemographic and structural conditions have consequences for the labor market participation of young persons with disabilities (YPWD) in vocational rehabilitation (VR). As the type of program determines the labor market chances, we analyze the processes of selecting active labor market programs (ALMP) in VR. Which factors determine the allocation to (1) programs in general and (2) moreover, the allocation to specific programs?

**Materials and methods:**

We conduct logistic (1) and multinomial regression (2) using register data of the German Federal Employment Agency. Besides variables on the micro level, we control for a wide range of structural and organizational influences. The sample comprises VR and employment biographies of 255,009 YPWD accepted to VR between 2010 and 2015. Program participation is restricted to start 180 days after VR acceptance.

**Results:**

Sociodemographic factors, like age and the status before entering VR as well as the local apprenticeship market as a structural condition, highly influence the general allocation to ALMP. For the allocation to specific ALMP, sociodemographics (age, education, type of disability, status before entering VR) are highly relevant. Furthermore, structural conditions (regional structure of subsidized vocational training and of the apprenticeship market as well as local work possibilities on a special labor market for PWD) and – to a lesser extent - re-organization processes at the FEA (NEO, VR cohort) are important determinants.

**Conclusion:**

(Automatic) paths into VR programs for especially persons with mental disabilities in sheltered workshop are clearly shown. Furthermore, it is somewhat questionable that YPWD participate more often in sheltered workshops in regions where sheltered work possibilities are more common, as well as where NEO was implemented locally; and participate more often in company-external vocational training where VR service providers are commissioned to a greater extent.

## Introduction

Health conditions and labor market participation are bidirectionally related [1, 2]. On the one hand, unemployment is associated with negative (psychological) health outcomes; on the other hand, persons with restricted health have a higher risk of losing their jobs, and their chances of reintegration are lower [3–5]. Furthermore, poor health reduces the probability of finding a job [6].

In addition, research has shown that there are multiple scarring effects resulting from unemployment at the very beginning of the school-to-work-transition. Unemployment experiences in younger life lead to multiple restrictions in later life such as an increasing number of unemployment periods or lower wages [7, 8]. If persons experience economic difficulties and manage to overcome them at an early stage they less often face a high-risk health trajectory [9].

Being unemployed in younger life also causes greater mental health problems both in the short and long term [10]. Cruces, Ham and Viollaz [11] illustrate those significant scarring effects of youth unemployment. The longer persons experience unemployment in youth, the worse they rate mental health in adulthood. Nygren, Gong and Hammarström [12] show that for women, unemployment in early life (between 16 and 21) is related to hypertension as an adult (around the age of 43). Morrell et al. [13] analyzed links between unemployment and psychological morbidity that accompany unemployment in young people, stating: “an effective cure of psychological morbidity resulting from unemployment is […] a job” (p. 1563). Furthermore, poor health during childhood aggravates labor market inequality during the life course [14]. Thus, specific support for a smooth school-to-work-transition will positively impact the integration and career quality.

In this paper, we focus on a vulnerable group: young persons with disabilities (YPWD) at their first transition into the labor market. Specifically, for disadvantaged young people with restricted health, this transition constitutes a critical point in their employment biography [15]. To improve labor market chances and to counteract potential scarring effects, vocational rehabilitation (VR) helps those YPWD find an occupational orientation and achieve an occupational degree. The latter is important, because in Germany an occupational degree is central for achieving labor market participation and thus, societal participation [16]. However, to date, there is a lack of empirical knowledge regarding the allocation process of YPWD into VR programs in Germany, especially regarding the following questions: *Which factors on various levels determine the allocation to (1) programs in general and (2) moreover, the allocation to specific programs*.

At the beginning of VR, the YPWD – the rehabilitant – and the VR counsellor decide together on the choice of active labor market programs (ALMP). Since it is possible and common to allocate several programs during the VR process (e.g., a vocational preparation and orientation phase followed by vocational training), we must select. We decided to look at the first program, as we assume that the first program refers to the overall VR strategy. Thus, if rehabilitants attend a prevocational training program as their first program, the counsellor very likely suspects a lack of vocational training maturity. A preparation phase is not necessary if the counsellor directly opts for vocational training. In our analysis, we consider that elements such as sociodemographic factors or the type of disability determine the probability of program participation.

We additionally assume that external (sociopolitical) structural conditions and institutions set a framework for activities, leading to differing allocation processes. We conduct multivariate logistic and multinomial regression analyses, using longitudinal administrative data from the German Federal Employment Agency (FEA) for cohorts of YPWD starting VR in 2010 through 2015.

## Background

To analyze the support structures for YPWD alleviating their labor market transition, we consider the German vocational training system, as well as the specifics of the VR process for YPWD.

### Vocational Training in Germany

A cornerstone of German vocational training is the so-called dual vocational training. It consists of a theoretical and a practical part. The practical part takes place in a company; the theoretical part in a vocational school [*Berufsschule*] and usually lasts for two or three years. Trainees receive apprenticeship pay. Characteristically, vocational training occurs in an unsubsidized in-firm manner. In Germany, a vocational training degree is especially important for a stable and sustainable participation in the labor market [15–17]. Additionally, after in-firm training the probability to remain in the company is higher. Takeover rates have increased in recent years, reaching 74% in 2020 [18]. In Germany, YPWD already seem to have more difficulties with the school-to-work transition because many are still educated within special schools where chances for receiving a (higher) schooling degree as well as labor market chances are comparatively low [19, 20]. As a result, they usually have lower educational and vocational qualifications compared to the general population [21]. Furthermore, their health situation or disability label might hinder their labor market transition. Generally, being male with higher education as well as having higher parental educational levels are promoting factors for successfully entering the labor market [22]. Additional factors promoting YPWD to enter the labor market are, e.g., “[…] high psychosocial level of functioning, low depression and high dispositional optimism […]” [22].

### VR Process for YPWD in Germany

To support this transition, the German welfare state provides VR including special ALMP for YPWD.

According to the German Social Code Book IX (Sect. 2 (1)) “a person has a disability if they have a physical, psychological, intellectual or sensory impairment that, in interaction with attitudinal and environmental barriers, is highly likely to impair their equal participation in society for longer than six months. A person has an impairment if their physical and health condition falls short of that typical for their age. A person is at risk of disability if such an impairment is to be expected.” [23].

Most often, YPWD in VR are most often recognized with a learning disability, a psychological, mental or physical disability [24].

The FEA is responsible for financing and promoting VR for almost all YPWD. If young people have health restrictions or a disability and it is therefore difficult for them to take up regular vocational training without further support, they can apply for VR. To provide necessary support and information on VR, VR counsellors from the FEA visit students from (special) schools, offering potential support within a path of well-marked avenues within the funding opportunities of the FEA. Thus, half of YPWD in VR enter VR directly after (special) school [24]. Another relevant group of YPWD find their way through vocational counselling and unemployment where they are redirected to the VR department at the agency; these YPWD have discovered difficulties in their school-to-work transition often due to psychological illnesses [20, 33].

Generally, PWD have the right to accept VR; however, they are not obliged to take the offer. Though the VR candidate has a legal right to VR, the counsellors‘ decision on whether to accept a person to VR, is based on different criteria that might also lead to not accept a candidate for VR: vocational aspirations and motivation of the YPWD, assessments carried out in advance (regarding their ability to work and potential tasks that can be carried out despite a disability), regional labor market conditions and their professional experience how the YPWD can find their way into the labor market and which programs are suitable and feasible for the candidate [25].

Ideally, VR counsellors have longstanding experiences in counselling PWD and based on this experience they (usually) know a lot about what works for whom. Of course, the counsellor takes different information into account when it comes to choosing a program: the motivation, interests and abilities of the YPWD, the range and availability of local programs or if boarding school accommodation (for programs outside the region/social background) is appropriate. The decision is made together with the YPWD. The VR counsellor can choose from various ALMP, pointing to different integration strategies for the individual need: The best case in terms of labor market integration chances would be the selection of an in-firm vocational training with work experience in a natural setting [26]. If YPWD cannot achieve regular in-firm training, supported programs range from subsidized vocational training – where the employer receives apprenticeship subsidies – to company-external vocational training, where both theoretical and practical parts of vocational training takes part in company-external environments – to training in sheltered workshops. Prevocational training programs are often necessary before training to identify individual strengths, achieve training maturity and to prevent drop-outs. Participation in such programs, however, might also be used to bridge waiting times, since vocational training always starts in the autumn of each year.

## Theoretical Reflections, Research Questions, Influencing Factors and Hypotheses

We are interested in the processes of selecting ALMP and associated strategies: *Which factors determine the allocation to (1) programs in general and (2) moreover, the allocation to specific programs.* In answering these questions, we focus on the capability approach [27, 28]. The aim of the capability approach is to explain social inequality by considering individual differences in realizing life chances. The extent to which capabilities can be improved, depends inter alia on ‘societal opportunities’ as part of socially determined opportunities [29, 30], e.g. the access to certain training programs. The approach emphasizes extending resources (e.g. material, social or cultural capital) and differentiates between functionings, conversion factors and capabilities. Functionings or skills, e.g. are individual characteristics and, in general, things that have already been achieved (e.g. graduation, health). Skills are, therefore, also characterized as actions (doings) or as states (beings). Capabilities are rather properties to realize certain chances (here: participation in VR programs). Finally, conversion factors are distinguished. They help people to seize opportunities and transform them into abilities in order to improve the realization of opportunities and reduce disabling conditions. They e.g. provide the infrastructure, despite difficulties in the training market, to gain training maturity and to undertake training. If YPWD succeed in completing vocational training, they can in turn improve their ability to participate in the labor market. Therefore, we analyze associations between conversion factors and capabilities as follows: We assume that special conditions at the labor and vocational training market and the organizational conditions of local employment agencies have consequences on the allocation of VR programs.

We use an analytical model to identify the main factors for the decision (1) to generally take part in ALMP and (2) to take part in a specific program. We assume that individual as well as structural factors are relevant.

As structural factors, we include (a) the *year of entering VR* and (b) the *regional structure of the apprenticeship market* (at the time of 2010) to approximate labor market conditions. Behind the latter lies a typology of regional training markets in Germany, showing that certain features like the transition rate into in-firm vocational training, the number of high-school graduates within the cohorts, the local unemployment rate etc. are important factors to characterize a region in terms of vocational training markets [31]. The typology ranges from living in a rural region with average apprenticeship market environment (AME) to living in a city with high employment rates. Furthermore, the *regional share of subsidized vocational training* (c) is captured by the local share of subsidized disability-specific vocational training (subsidized in-firm training, company-external training) versus regular in-firm vocational training. It represents the local possibilities YPWD have in one program or another. We assume that due to a local offer of subsidized and company-external training, YPWD are more often allocated to company-external programs. We also consider *local work possibilities on a special or second labor market for people with disabilities* (d). This indicator compares the number of people working in subsidized employment within sheltered workshops, disability-specific vocational training centers [*Berufsbildungswerke*], disability-specific vocational promotion centers [*Berufsförderungswerke*] or integrative companies [*Integrationsfirmen*, pursuing economic interests while 25 to 50% of the employees are persons with disabilities] to the number of regular employees. By using this indicator, we assume that the higher the local share of a special labor market, the more people are allocated to those special VR programs.

Additionally, we assume that one institutional factor has an influence on the allocation process: (f) the *re-organization of the local employment agencies* (of the FEA) in 2012, called NEO [*Neuorganisation der Bundesagentur für Arbeit*]. The aim of the FEA was a harmonization of political territorial (regional) structures (administrative regions or districts) with organizational (FEA-internal) territorial structures and a further development of internal organizational structures [32]. To achieve this, nearly 30% of all agencies in our dataset were affected by such a re-organization. Connected to this, we assume that there are different regional practices in promoting certain programs or VR service providers, depending on whether an agency was reorganized or not. This assumption is based on the fact that the agency was not only (territorially) re-organized (e.g. smaller agencies were merged; bigger agencies gave territorial responsibilities away) but also internally restructured. Tasks were redistributed within the organization. This could have led to the separation of teams that had previously worked well together, or to specialists being assigned new complex tasks - such as VR. This might have led to uncertainties and might have influenced the allocation to VR programs. Thus, some counsellors started in the quite complicated field of VR without prior experience. Therefore, they may need a period of orientation, where they might stick to their former toolkits for ALMP. This would result in the provision of more general and less disability-specific programs.

Furthermore, we assume that individual characteristics of the YPWD may influence allocation. Included are (g) *sociodemographic factors*, (h) the *type of disability* and (i) the *status before VR*. We assume that higher age, lower educational qualifications and psychological/mental disabilities as well as coming from a special school or having no prior status hinder YPWD entering vocational training directly or taking up in-firm vocational training as well as foster the allocation to sheltered workshops and to take up any ALMP. Especially, for persons with mental disabilities automated pathways are often described in the literature [20, 33]; furthermore, persons with psychological disabilities are often said to need a mental stabilization phase first in order to find their occupational career path [31].

## Data and Methods

### Data

Analyses are based on a dataset collected within the administrative process of VR covered by the FEA (German Reha-Process Data Panel [RehaPro]) [33]. It comprises all persons applying for VR at the FEA. The data includes inter alia sociodemographic information such as sex, age, nationality, place of residence on the district level, schooling degree, as well as the type of primary disability mainly affecting labor market participation. On a daily basis, the dataset provides detailed information on employment history, spanning retroactively from first contact with the FEA (e.g., vocational counselling), or the first employment notification to the social security system and up to the end of 2015. It also comprises information on participation in general programs available for all unemployed persons and disability-specific ALMP (available only for rehabilitants) and is enriched with data on the complete VR process. The dataset allows differentiation of the programs in type, order and duration during VR. By using the place of residence, the local vocational training market can be identified, as well as the local employment agency and if it was affected by NEO. The rehabilitants included in the analyses were officially recognized as rehabilitants by the FEA in the years 2010 to 2015.

### Methods and Materials

Based on logistic regression, in the first step, we analyze general participation in ALMP after being officially accepted by the FEA as a vocational rehabilitant. In the second step, based on a multinomial regression for those participating in ALMP, we want to know which factors are associated with the allocation to specific programs.

The data on program participation is right-censored until April 2016. In order to approach this censoring, we restrict the program allocation to be finished during the first 180 days after a person is accepted to VR. Thus, programs starting after 180 days after VR acceptance are counted as “no program participation”.

*Dependent Variables*. The first main outcome is the general *participation in programs*. It includes participation in any ALMP during VR (yes (1) or no (0)). The second main outcome is the *type of the first allocated program* with the following categories: (a) (un)subsidized in-firm vocational training, (b) company-external vocational training, (c) general prevocational training program (e.g., insights into various occupational fields, achieving lower secondary schooling degree), (d) disability-specific prevocational training programs (analogous with the preceding, but with additional disability-specific program characteristics, e.g., in specific VR centers providing additional medical and/or psychological care), (e) other prevocational training (e.g., aptitude assessment, entry qualifications), (f) training in sheltered workshops and (e) other programs.

*Independent Variables*. As described above, we consider different independent variables: *sex*^1^, *age* (at the beginning of VR), *nationality* (German vs. non-German), *highest schooling degree* (no schooling degree, lower secondary schooling degree, intermediate secondary schooling degree, higher secondary schooling degree), *type of disability* (psychological, mental, musculoskeletal, learning and other disabilities) and *status before entering VR* (unemployed, vocational guidance, contributed employment, vocational or other school type, regular school, special school, prevocational programs, other programs, no prior status). *Re-organization of the local employment agency* (NEO) is a dummy variable. *Year of entering VR* ranges from 2010 to 2015, *Regional structure of apprenticeship market* contains ten categories. The variables *Regional structure of subsidized vocational training*, and *local work possibilities on a special or second labor market for people with disabilities* are dummy variables distinguishing local agencies that are above or below the German average.

## Results

### Description of the Population

The characteristics of YPWD entering VR in 2015 are shown in Table 1a. Six of ten participants were men; four of ten were women. Almost half of the population had a learning disability, followed by psychological (22%) and mental disabilities (17%). Over half of the population was between 17 and 20 years old (56%). This more or less corresponds to the general school graduation age in Germany (lower secondary schooling degree: 16.6 years of age, intermediate secondary schooling degree: 17.1 years of age [34]). 45% finished school with a lower secondary schooling degree; 36% finished school without a schooling degree. In the overall German population, only 3.7% have no schooling degree. The YPWD often come from a special school: 31% entered VR directly after special school; 20% came from regular or other schools. 32% were unemployed or had been to vocational guidance before VR.

**Table 1:** **a**: *Description of the Population - Characteristics of YPWD starting VR in 2015 (independent variables), N = 41,199*

		Share	
Participant sex			
	Male	61	%
	Female	39	%
Participant age			
	Age under 17	22	%
	Age 17 to 20	56	%
	Age 21 to 24	15	%
	Age 25 or older	7	%
Participant highest schooling degree			
	No (general) schooling degree	36	%
	Lower secondary schooling degree	45	%
	Intermediate secondary schooling degree	14	%
	Higher secondary schooling degree	4	%
	Missing value	1	%
Participant type of disability			
	Psychological	22	%
	Learning	48	%
	Mental	17	%
	Musculoskeletal	5	%
	Others	7	%
Participant status before entering VR			
	Vocational guidance	11	%
	Unemployed	21	%
	Contributed employment	7	%
	Regular school	6	%
	Vocational school or other school types	31	%
	Special school	14	%
	Prevocational training program	3	%
	Other programs	5	%
	No prior status	3	%
Regional structure of apprenticeship-market			
	Rural districts with highly developed industrial sector	8	%
	Rural, average AME	7	%
	City, advantageous AME, little competition	9	%
	Urban, strong industrial environment	7	%
	Urban, average conditions	13	%
	Rather urban, low unemployment, strong competition	10	%
	City, high unemployment	11	%
	Rather urban, advantageous AME	15	%
	Rural, developed secondary sector, strong competition	14	%
	Rural, little industrial environment, strong competition	5	%
Participant nationality			
	German	93	%
	Non-German	7	%
Regional structure of subsidized vocational training			
	Local share below average	67	%
	Local share above average	33	%
Local work possibilities on a special or second labor market for people with disabilities			
	Local share below average	53	%
	Local share above average	47	%
Re-organization of local employment agencies (NEO)			
	No	72	%
	Yes	28	%

AME: Apprenticeship market environment; NEO: re-organization of the local employment agencies [Neuorganisation der Bundesagentur für Arbeit]; VR: Vocational Rehabilitation, Data Source: RehaPro, own calculation

Looking at the first dependent variable – the general participation in programs -, we find that every year 20 to 25% did not participate in any VR program during the first 180 days after VR acceptance. When we look at the second dependent variable - at the type of program -, we find that most of them participated in disability-specific prevocational training programs. These numbers slowly increased from 24% to 2010 to 32% in 2015. The second most frequent first program chosen were sheltered workshop programs (as a preparation for employment in sheltered workshops) with 22% in 2010 and 20% in 2015; about 13% of rehabilitants start directly with company-external vocational training (Table 1b). Only one in ten participated directly in in-firm vocational training. The participation in general prevocational training programs as a first program decreased during the observation period from 15 to 8% (Table 1b).

**Table 1:** **b**: *Descriptives of the Population, dependent variables by year of entering VR*

	Allocation into labor market programs (1)			Allocation into… (2)							
Year of entering VR	no	yes	N	(un)subsidized in-firm vocational training	company-external vocational training	general prevocational training	disability-specific prevocational training	other prevocational training	training in sheltered workshop	other labor market programs	N
2010	24%	76%	44,819	11%	14%	15%	24%	8%	22%	6%	34,088
2011	21%	79%	44,083	11%	14%	13%	25%	9%	21%	7%	34,653
2012	21%	79%	42,691	10%	13%	12%	27%	10%	21%	7%	33,617
2013	20%	80%	41,103	10%	13%	9%	29%	10%	20%	8%	32,789
2014	21%	79%	41,114	9%	13%	8%	31%	9%	21%	8%	32,655
2015	25%	75%	41,199	9%	11%	8%	32%	10%	20%	8%	30,962
	56,245	198,764	255,009								198,764

VR: Vocational Rehabilitation, Data Source: RehaPro, own calculation

### Model 1: Logistic Regression: General Participation in Programs mainly associated with Sociodemographics and the Regional Apprenticeship Market

In the first model, we observed group differences in the general participation in programs during the VR process (Table 2). The results show that although more men than women took part in VR, men had slightly fewer chances to participate in programs. Furthermore, persons with German nationality, persons between 17 and 24 years and persons with a diploma were more likely to participate in programs. Looking at the type of disability, persons with a mental disability had the highest participation probability compared to those with other disabilities. The status before the acceptance as a rehabilitant also played a central role and is highly influential in explaining variances: YPWD coming from regular or special schools were the least likely to participate in a program 180 days after acceptance as vocational counselling often takes place early before school ends; thus, it is possible that acceptances also take place already during the last school year. Those who already took part in general ALMP are the most likely to take up a program. Here, the need for a disability-specific program has been recognized during program participation.

**Table 2:** *Rehabilitants’ Allocation into Programs in General (yes/no), logistic regression*

Covariates		Model 1: Allocation into labor market programs yes/no	
		ame/sig. (s.e.)	
Participant sex (Ref.:° Female)			
	Male	-0.02	*** (0.00)
Participant age (Ref.:° under 17 years)			
	Age 17 to 20	0.07	*** (0.00)
	Age 21 to 24	0.04	*** (0.00)
	Age 25 or older	0.00	(0.00)
Participant nationality (Ref.:° non-German)			
	German	0.03	*** (0.00)
Participant highest school graduation (Ref.:° no schooling degree)			
	Lower secondary schooling degree	0.03	*** (0.00)
	Intermediate secondary schooling degree	0.03	*** (0.00)
	Higher secondary schooling degree	0.02	*** (0.00)
Participant type of disability (Ref.:° psychological)			
	Learning	0.02	*** (0.00)
	Mental	0.04	*** (0.00)
	Musculoskeletal	0.00	(0.00)
	Others	0.01	+ (0.00)
Participant status before entering VR (Ref.:° vocational guidance by the FEA)			
	Unemployed	-0.05	*** (0.00)
	Contributed employment	-0.01	* (0.00)
	Vocational school or other school type	-0.07	*** (0.00)
	Regular school	-0.14	*** (0.00)
	Special school	-0.16	** (0.00)
	Prevocational program	0.08	*** (0.01)
	Other programs	0.23	*** (0.01)
	No prior status	-0.05	*** (0.01)
Regional structure of apprenticeship market (Ref.:° rural districts with highly developed industrial sector)			
	Rural, average AME	-0.04	*** (0.00)
	City, advantageous AME, little competition	-0.02	*** (0.00)
	Urban, strong industrial environment	0.02	** (0.00)
	Urban, average conditions	-0.03	*** (0.00)
	Rather urban, low unemployment, strong competition	0.04	*** (0.00)
	City, high unemployment	-0.04	*** (0.00)
	Rather urban, advantageous AME, average competition	0.03	*** (0.00)
	Rural, developed secondary sector, strong competition	0.04	*** (0.00)
	Rural, little industrial environment, strong competition	0.09	*** (0.01)
Regional structure of subsidized vocational training (Ref.:° local share below average)			
	Above average	0.03	*** (0.00)
Local work possibilities on a special or second labor market for people with disabilities (Ref.:° local share below average)			
	Above average	0.01	*** (0.00)
Re-organization of the local employment agencies (Ref.: NEO marker: no)			
	Yes	0.01	*** (0.00)
Year of entering VR (Ref.:° 2010)			
	2011	0.02	*** (0.00)
	2012	0.03	*** (0.00)
	2013	0.03	*** (0.00)
	2014	0.03	*** (0.00)
	2015	-0.01	** (0.00)
Number of rehabilitants		252,032 (2,977 missing values)	
Pseudo-R2		0.0599	

AME: Apprenticeship market environment; NEO: re-organization of the local employment agencies [Neuorganisation der Bundesagentur für Arbeit; VR: Vocational Rehabilitation

Data Source: RehaPro, own calculations; sig. = significance level: + p < 0.1, * p < 0.05, ** p < 0.01, ***p < 0.001, 95% confidence interval, ame = average marginal effects, s.e. = standard error]; Ref.:°: Reference category

The local vocational training market affects the probability to take part in VR programs as well. Those persons who live in regions where there is strong competition on the local vocational training market show the highest chances to participate in a program, especially those who live in rural regions with little industrial environment.

For persons living in regions where the regional share of subsidized vocational training was higher, the participation probability was higher as well. Low, but significant differences in program participation can also be observed for regions where NEO has affected the local employment agency and for regions where work possibilities on a special labor market for PWD are above average. Here, YPWD slightly more often take part in programs. Finally, there are small group differences for the year of VR acceptance. YPWD starting VR in 2015 are less likely to start a program in comparison to all prior cohorts.

### Model 2: Multinomial Regression: Local Labor Market and Sociodemographics as Influencing Factors Regarding the Participation in Specific Programs

In the second model, we observed allocation mechanisms into different types of programs, running a multinomial regression (Table 3). Hence, we restricted the analysis to those who participated in programs during the first 180 days after VR acceptance. Age and education played a major role. Persons under 17 years of age had the highest probability of being allocated into prevocational training programs (general or disability-specific) and into in-firm vocational training; older persons most often went into company-external vocational training or – especially persons aged 25 or older – into sheltered workshops. Persons without a schooling degree had the highest probability to enter sheltered workshops. YPWD with a higher secondary schooling degree went directly into (in-firm) vocational training.

**Table 3:** *Rehabilitants’ Allocation into Specific Programs, Multinomial Regression*

Covariates		Model 2: Allocation into…													
		(un)subsidized in-firm vocational training		company-external vocational training		general prevocational training		disability-specific prevocational training		other prevocational training		Training in sheltered workshop		other labor market programs	
		ame/sig. se		ame/sig. se		ame/sig. se		ame/sig. se		ame/sig. se		ame/sig. se		ame/sig. se	
Participant sex (Ref.:° Female)															
	Male	-0.02	***	0	*	0.01	***	0.02	***	-0.01	***	0	***	-0	***
		0		0		0		0		0		0		0	
Participant age (Ref.:° under 17 years)															
	Age 17 to 20	-0.04	***	0.03	***	-0.01	**	-0.07	***	-0		0.07	***	0.02	***
		0		0		0		0		0		0		0	
	Age 21 to 24	-0.07	***	0.08	***	-0.04	***	-0.18	***	0.04	***	0.11	***	0.05	***
		0		0		0		0		0		0		0	
	Age 25 or older	-0.09	***	0.09	***	-0.09	***	-0.26	***	0.07	***	0.19	***	0.09	***
		0		0		0		0		0		0		0	
Participant nationality (Ref.:° non-German)															
	German	0.02	***	0.01	*	-0.01	**	-0.01	**	-0		-0.01	+	-0	
		0		0		0		0		0		0		0	
Participant highest schooling degree (Ref.:° no schooling degree)															
	Lower secondary schooling degree	0.05	***	0.06	***	-0.01	***	-0.01	***	0.01	***	-0.1	***	-0.02	***
		0		0		0		0		0		0		0	
	Intermediate secondary schooling degree	0.10	***	0.06	***	-0.01	***	-0.05	***	0.05	***	-0.14	***	-0.01	***
		0		0		0		0		0		0		0	
	Higher secondary schooling degree	0.15	***	0.04	***	-0.03	***	-0.07	***	0.07	***	-0.17	***	0.02	***
		0		0		0		0.01		0		0		0	
Participant type of disability (Ref.:° psychological)															
	Learning	0.06	***	0.11	***	0.14	***	-0.05	***	-0.09	***	-0.18	***	0.01	***
		0		0		0		0		0		0		0	
	Mental	-0.01	***	-0.07	***	-0.03	***	-0.31	***	-0.06	***	0.43	***	0.06	***
		0		0		0		0		0		0		0	
	Musculoskeletal	0.14	***	0.03	***	0.02	***	-0.18	***	-0.00		-0.07	***	0.07	***
		0		0		0		0.01		0		0		0	
	Others	0.08	***	0.05	***	0.02	***	-0.11	***	0.02	***	-0.08	***	0.01	***
		0		0		0		0		0		0		0	
Participant status before entering VR (Ref.:° vocational guidance by the FEA)															
	Unemployed	-0.01	***	-0.02	***	-0.02	***	-0.05	***	0.02	***	0.03	***	0.05	***
		0		0		0		0		0		0		0	
	Contributed employment	0.22	***	-0.02	***	-0.05	***	-0.15	***	-0.01	***	-0.04	***	0.05	***
		0		0		0		0		0		0		0	
	Vocational school or other school type	0.02	***	0.06	***	-0.04	***	-0.06	***	0		-0		0.02	***
		0		0		0		0		0		0		0	
	Regular school	0.01	***	-0.06	***	-0.03	***	0.03	***	0.02	***	0.01	+	0.03	***
		0		0		0		0.01		0		0		0	
	Special school	0.03	***	-0.06	***	-0.04	***	-0.03	***	0.01	***	0.08	***	0.01	***
		0		0		0		0		0		0		0	
	Prevocational program	0.08	***	0.04	***	0.08	***	-0.16	***	0.02	***	-0.07	***	0.01	
		0		0		0		0		0		0.01		0	
	Other programs	0.1	***	-0.01	*	-0.11	***	-0.30	***	-0.07	***	0.12	***	0.28	***
		0		0		0		0		0		0		0	
	No prior status	0		-0.08	***	-0.04	***	-0.06	***	0.02	***	0.15	***	0.01	*
		0.01		0.01		0.01		0.01		0		0		0	
Regional structure of apprenticeship market (Ref.:° rural districts with highly developed industrial sector)															
	Rural, average AME	-0.02	***	-0.03	***	-0.02	***	0.07	***	-0		0.01	*	-0	+
		0		0		0		0		0		0		0	
	City, advantageous AME, little competition	-0.01	**	-0.02	***	0		0.05	***	-0.06	***	0.04	***	0	
		0		0		0		0.01		0		0		0	
	Urban, strong industrial environment	0.01	***	-0.01		0.08	***	-0.05	***	-0.09	***	0.05	***	0.01	+
		0		0		0		0		0		0		0	
	Urban, average conditions	-0		-0.02	***	0.11	***	-0.07	***	-0.07	***	0.04	***	0.01	*
		0		0		0		0		0		0		0	
	Rather urban, low unemployment, strong competition	0.04	***	0.06	***	0.01	***	-0.08	***	-0.07	***	0.02	***	0.01	***
		0		0		0		0		0		0		0	
	City, high unemployment	-0.04	***	-0.04	***	0.03	***	0.05	***	-0.04	***	0.05	***	-0.01	***
		0		0		0		0		0		0		0	
	Rather urban, advantageous AME, average competition	0.03	***	-0.01	**	0.02	***	-0.02	***	-0.07	***	0.04	***	0.01	***
		0		0		0		0		0		0		0	
	Rural, developed secondary sector, strong competition	0.05	***	0.02	***	0.01	***	-0.06	***	-0.07	***	0.03	***	0.01	***
		0		0		0		0.01		0		0		0	
	Rural, little industrial environment, strong competition	0.04	***	-0.01		-0.03	***	0.01		-0.05	***	0.04	***	0.01	*
		0		0		0		0.01		0		0		0	
Regional structure of subsidized vocational training (Ref.:° local share below average)															
	Above average	0.01	***	0.08	***	-0.04	***	-0.02	***	-0.01	***	-0.01	***	0	*
		0		0		0		0		0		0		0	
Local work possibilities on a special or second labor market for people with disabilities (Ref.:° local share below average)															
	Above average	-0.03	***	-0.02	***	0.05	***	0		-0.02	***	0.03	***	-0.01	***
		0		0		0		0		0		0		0	
Re-organization of local employment agency (Ref.: NEO marker: no)															
	Yes	-0.03	***	-0.02	***	-0.01	***	-0.04	***	-0.01	***	0.01	***	-0.01	***
		0		0		0		0		0		0		0	
Year of entering VR (Ref.:° 2010)															
	2011	0		-0		-0.01	***	0.01	***	0.01	***	-0.01	***	0.01	***
		0		0		0		0		0		0		0	
	2012	-0.01	**	-0		-0.02	***	0.03	***	0.01	***	-0.02	***	0.01	**
		0		0		0		0		0		0		0	
	2013	-0.01	***	-0		-0.04	***	0.06	***	0.01	***	-0.03	***	0.01	***
		0		0		0		0		0		0		0	
	2014	-0.02	***	-0.01	*	-0.05	***	0.08	***	0.01	***	-0.03	***	0.02	***
		0		0		0		0		0		0		0	
	2015	-0.03	***	-0.01	***	-0.05	***	0.09	***	0.01	***	-0.03	***	0.02	***
		0		0		0		0		0		0		0	
Number of rehabilitants		198,061 (703 Missing values)													
Pseudo-R2		0.286													

AME: Apprenticeship market environment; NEO: re-organization of the local employment agencies [Neuorganisation der Bundesagentur für Arbeit]; VR: Vocational Rehabilitation

Data Source: RehaPro, own calculations; sig. = significance level: + p < 0.1, * p < 0.05, ** p < 0.01, ***p < 0.001, 95% confidence interval, ame = average marginal effects, s.e. = standard error; Ref.:°: Reference category

The type of disability was also a key factor. Young persons with psychological illnesses had comparatively higher chances of receiving disability-specific prevocational training. In addition, persons with mental disabilities went most likely into sheltered workshop programs. In contrast, persons with musculoskeletal disabilities had the highest chances of receiving in-firm vocational training directly.

The status before acceptance was important in the allocation process as well. YPWD coming from employment or from general ALMP into VR took part in prevocational training less frequently; except for those from general prevocational programs, they also most often took part in disability-specific prevocational programs afterwards, as it might have become apparent that disability-specific arrangements were necessary for them. Those employed at the time of VR acceptance most often directly received subsidized in-firm vocational training. And finally, those without a prior status, as well as those from a special school went most likely into sheltered workshops. Somehow differently to what we formulated as an expectation, rehabilitants being promoted by local employment agencies that were affected by NEO slightly more often went to sheltered workshops. Though, this is a disability-specific program, it is well-known to non-VR counsellors. This is supported by the results from the year of entering VR: compared to those entering VR in 2010, rehabilitants in later cohorts participated less often in sheltered workshops and general prevocational programs and more often in disability-specific and other prevocational training programs – especially after 2012 when NEO was implemented at the FEA.

The local apprenticeship market shows partly high effects but sometimes vague allocation patterns. However, one tendency is that in urban regions YPWD most often participate in general prevocational training; this is also true for the participation in sheltered workshops, YPWD less likely seemed to participate in sheltered workshops when they lived in rural areas. In this respect, other information on the local labor market is even more striking: if the local share of subsidized vocational training is above the national average, the chances for participation in company-external vocational training are higher. Thus, the very existence of these organizations in the local area of the employment agency seems to be a decisive factor in the allocation process. Furthermore, if the indicator for local work possibilities on a special or second labor market for PWD is above average – thus more sheltered workshops and integration firms are nearby -, YPWD more likely participate in general prevocational training programs, as well as sheltered workshops.

## Strengths and Limitations

Before discussing our results, strengths and limitations should be mentioned. Starting with the latter, first, we were unable to identify and describe the impact of health restrictions on training and employment. These can differ significantly, even if the type of disability is the same. Second, the type of disability was recorded only in a highly aggregated form, and moreover, only the main disability was documented. Multimorbidity was not recorded. This also may influence the allocation process. Therefore, we are not able to identify mediating pathways.

The main strengths of the study are the full data sample as well as the longitudinal design of the data and comprehensive information regarding structural and organizational variables at the local level. Since the data comprise the basic population of all rehabilitants from the FEA, representative statements e.g., about push and pull factors of allocation practices into ALMP are possible. The inclusion of institutional aspects and local labor market conditions enhances the informative value.

We do not explain much variance in the general allocation process to ALMP (R^2^ 0.06). However, the model for the determination of the choice of specific programs has a high level of explanatory power (Pseudo R^2^ 0.29). This shows that the selection of specific programs can be explained by the data at hand, whereas the general take-up of programs is determined by additional unobserved factors.

## Discussion

Based on the Capability Approach, we assumed that certain functionings (like sociodemographics) and conversion factors (like external structural conditions as the regional structure of subsidized vocational training) hinder or promote YPWD in their capability to achieve (specific) programs. The results partly support our assumptions. We provide empirical evidence for the association between functionings as well as conversion factors and outcomes in order to explain general and primary allocation into VR programs. Specifically, we find that the general allocation to programs is mainly associated with sociodemographic factors – except for one conversion factor: the local apprenticeship market. When looking at the specific allocation to ALMP, sociodemographics are central determinants as well, but in addition, we find that some of the conversion factors – structural factors – play an important role as well.

Hereinafter, the most influential and surprising results from above will be discussed. The status before entering VR is the most influential factor when it comes to the selection to any program: if people enter VR from other (general) programs, they most likely take up any program, as they already showed willingness to invest in their qualification; however, they take up programs less often if they were in school at the time of VR acceptance. However, this effect is due to the fact that we tackle the censoring of the data by restricting programs to begin during the first 180 days after VR acceptance. As we did not have that effect without this restriction, we might conclude that YPWD accepted for VR while still in school take longer than 180 days to start their first program. Furthermore, the local training market is relevant as a structural information: if competition among school graduates for training is higher and if YPWD live in rural regions with little industrial environment, the higher the probability to take up any program. This might be due to the circumstance that in these regions YPWD have less alternatives to VR.

A decisive factor in the choice of program is education: persons without a schooling degree most often go into sheltered workshops, while YPWD with higher secondary schooling degree go directly into vocational training. Therefore a (higher) schooling degree serves as a sign for the ability to master (regular) vocational training and the lack of a degree makes sheltered environments as a last resort for inclusion necessary.

Connected to this, the selection into ALMP is highly determined by the type of disability. As expected, persons with mental disabilities, frequently coming from special schools where the probability for (higher) educational degrees is lower [15], most often take part in programs in general and, more specifically, in sheltered workshops. However, if people go into sheltered workshops, their path is predefined for sheltered employment [21, 35] and there are very low chances of getting into the first labor market from there [36]. Furthermore, young persons with psychological disabilities are most often allocated into disability-specific prevocational training programs. They seem to need more orientation and stabilization than others. Therefore, there are specific VR service providers: besides prevocational training, these providers offer medical support as well, in order to prepare them for vocational training and the labor market [33].

Structural and organizational-specific conditions additionally influence the allocation processes to a certain extent. The year of entering VR has a significant influence; more recent cohorts have a higher probability of entering disability-specific and less often general prevocational training as well as sheltered workshops. Especially the former might reflect strategies in preventing later drop-outs, or a higher need of YPWD to receive disability-specific support during occupational orientation. Furthermore, this result and the results on the NEO variable support our assumption of a socialization period for the counsellors in the field of VR. Especially in the complex field of VR, the professional knowledge of the counsellors is crucial and should be trained as soon and as comprehensive as possible. The more time the counsellors have to become familiar with their new field of action (VR), the more their program allocation changes from general to disability-specific (prevocational) ALMP, and YPWD are less often allocated to sheltered workshops. The latter can be considered a one-way ticket to the secondary labor market as well as an easy way for the VR counsellor to close a case.

Finally, our results show that the selection into sheltered workshops is slightly more often observed in regions where work possibilities on a special labor market for PWD (like sheltered workshops) are above average. And the selection into company-external vocational training is higher in regions where subsidized vocational training is above average. This is interesting as it raises questions about whether the regional demand regulates the supply for vocational rehabilitants or whether it is the other way around.

## Conclusion

As a conclusion, we find certain predefined paths into different VR programs. Of course, capabilities as sociodemographic factors and conversion factors determine certain pathways. However, institutional and structural factors are also relevant in determining certain paths in non-inclusive environments, and that is at least questionable. Most rehabilitants need special support and many share a lack of vocational training maturity. For them, it might be the only way to achieve a vocational degree. However, that does not have to be true for all YPWD in VR. The type of disability does not necessarily reflect a reduced capability in employment activities or tasks. Therefore, an allocation into programs should depend mainly on the prerequisites of the individual person. Longstanding experience and a comprehensive training of the VR counsellor is crucial in this respect.

There is a need for VR as a sociopolitical instrument to counteract excluding tendencies on the labor market and to constitute the basis for building self-esteem and self-reliance and leading to a self-determined life with sufficient income. But following predefined paths and organizational factors, and on local availabilities and demands, VR may lead to social exclusion for some as an unintended consequence.

Avoiding this is not only important for the individual. The consequences for society must also be considered. Switching from an individual to a societal perspective shows that labor market participation is not only an individual value but is also important for labor market development in general. Current German labor market challenges include a rising average age of the overall population followed by shrinking labor force potential [37]. Even large influxes of refugees since 2015 in Germany will not change the situation effectively to stop this demographic development. Consequently, Germany is facing the necessity of increasing its labor force potential in many different ways. In this context, the course for the future is already partly displayed; the official retirement age was raised to 67, the participation rates for women were increased significantly. Here today, Germany has one of the top positions in Europe [38]. Participation rates for elderly persons were increased; 77% of the 55 to 59-year-olds are employed and almost half of the 60 to 64-year-olds are employed [39]. One further step toward altering the demographic change is to increase labor market participation of PWD and to counteract exclusion tendencies.

## Data Availability

The data that support the findings of this study are particularly protected social data. According to § 75 Book X of the German Social Code, the data are available on request from The Federal Ministry of Labor and Social Affairs via the Institute for Employment Research (department DIM). Data can only be analysed at the Institute for Employment Research or one of its locations outside Germany.

## Footnotes

1 We estimated separate models for men and women. However, differences were so small that we returned to use a dummy variable for sex within the models.
